# Autophagy mediates glucose starvation-induced glioblastoma cell quiescence and chemoresistance through coordinating cell metabolism, cell cycle, and survival

**DOI:** 10.1038/s41419-017-0242-x

**Published:** 2018-02-12

**Authors:** Lian Wang, Zhouchun Shang, Yang Zhou, Xinyu Hu, Yihong Chen, Yantao Fan, Xiaoyu Wei, Liang Wu, Qiujuan Liang, Jun Zhang, Zhengliang Gao

**Affiliations:** 1 Shanghai Tenth People’s Hospital, Tongji University School of Medicine, Shanghai, 200072 China; 2 Department of Regenerative Medicine, Tongji University School of Medicine, Shanghai, 200092 China; 3 BGI-Shenzhen, Shenzhen, China; 4 China National GeneBank, BGI-Shenzhen, Shenzhen, China; 5 Department of Cardiology, Shanghai Changzheng Hospital Second Military Medical University, Shanghai, China; 6 Advanced Institute of Translational Medicine, Tongji University School of Medicine, Shanghai, China, Shanghai, China

## Abstract

Metabolic reprogramming is pivotal to sustain cancer growth and progression. As such dietary restriction therapy represents a promising approach to starve and treat cancers. Nonetheless, tumors are dynamic and heterogeneous populations of cells with metabolic activities modulated by spatial and temporal contexts. Autophagy is a major pathway controlling cell metabolism. It can downregulate cell metabolism, leading to cancer cell quiescence, survival, and chemoresistance. To understand treatment dynamics and provide rationales for better future therapeutic strategies, we investigated whether and how autophagy is involved in the chemo-cytotoxicity and -resistance using two commonly used human glioblastoma (GBM) cell lines U87 and U251 together with primary cancer cells from the GBM patients. Our results suggest that autophagy mediates chemoresistance through reprogramming cancer cell metabolism and promoting quiescence and survival. Further unbiased transcriptome profiling identified a number of clinically relevant pathways and genes, strongly correlated with TCGA data. Our analyses have not only reported many well-known tumor players, but also uncovered a number of genes that were not previously implicated in cancers and/or GBM. The known functions of these genes are highly suggestive. It would be of high interest to investigate their potential involvement in GBM tumorigenesis, progression, and/or drug resistance. Taken together, our results suggest that autophagy inhibition could be a viable approach to aid GBM chemotherapy and combat drug resistance.

## Introduction

Cancer cells, as they develop and evolve, undergo metabolic reprogramming to sustain their rapid growth and proliferation. Thus, cancer cells often have distinct nutrient requirements such as higher level of glucose, a phenomena associated with Warburg effect which is characterized by high glycolytic rate and lactate production even if O_2_ is plentiful^1^^–^^3^. Dietary restriction and therapy, e.g., ketogenic diet (KD) of high fat and low carbohydrate, have been widely proposed and tested to starve and treat cancers^4,5^. Nonetheless, tumors cannot be simply regarded as a bulk of proliferating cells. They comprise heterogeneous populations of cells with metabolic activities dynamically modulated by spatial and temporal contexts^6,7^. Clearly, there are limitations to targeting specific metabolic pathways^8–10^.

The realization of inter and intra-tumor heterogeneity and the discovery of tumor stem cells is a major leap in cancer biology^6,11^. Tumors display elevated rates of glucose uptake and metabolism to sustain their rapid growth. But these demands are often not fully met and nutrient deprivation may cause subsets of cells to undergo enhanced autophagy and transition to quiescence^11,12^. Meanwhile, uncontrolled proliferation results in an acidic microenvironment lack of sufficient oxygen and nutrients, creating a safe haven for these slow dividing and sometimes, dormant cancer cells often of stem cell-like properties^13^. Radio- and chemotherapies are cytotoxic, relying on DNA replication and cell division. As such, slow dividing and/or entering quiescence is an effective way to evade therapies, incurring drug resistance and relapse^14,15^. Therefore, approaches capable to reactivate dormant cancer cells are a logical step to eradicate them and combat drug resistance.

Autophagy downregulates cancer cell metabolism, leading to quiescence and survival, and as such constitutes a vital mechanism of drug resistance^12,16^. Theoretically, autophagy inhibition should prevent tumor cell from entering quiescence and exert synergic effects with radio- and chemotherapies^17^. Following this logic, there are a number of ongoing clinical trials^18,19^. However, on the other hand, enhanced autophagy hinders cell growth and proliferation, slowing down tumor progression. Excessive autophagy, a self-eating mechanism, can even cause massive turnover of proteins and organelles, and have the potential to kill cancer cells^17^. Rapamycin, an inhibitor of oncogene *mTOR* and inducer of autophagy, has indeed been explored as a cancer treatment reagent^20,21^. Given the complexities, a better understanding of autophagy in concerned tumor settings shall help discern the role of autophagy in given tumors and develop effective combinatorial treatment, preventing resistance and relapse.

Glioblastoma (GBM) is the most lethal brain tumor with a median survival time of less than 18 months^22,23^. The mainstay treatment is surgical resection frequently with radio- and chemo- therapies^24^. Temozolomide (TMZ) is the standard chemotherapeutic drug for advanced GBM but often becomes ineffective with fast emerging resistance^8, 25,26^. KD have been suggested for GBM treatment with several clinical trials including KD as an adjuvant^4,5^. These approaches seem effective to many tumors and could prolong GBM patient survival^27^. Nonetheless, cancer recurrence and metastasis are often inevitable, leading to eventual therapeutic failure and morbidity.

In the present study, we have utilized two commonly used GBM cell lines U87 and U251 together with primary cancer cells from patients and sought to investigate whether and how autophagy is involved in GBM chemoresistance. Our results suggest that autophagy incurs chemoresistance through inducing cancer cell quiescence and survival. Autophagy manipulation can potentially aid chemotherapies and combat drug resistance.

## Results

### Glucose starvation sensitizes glioblastoma cells to chemotherapies

**Fig. 1:**
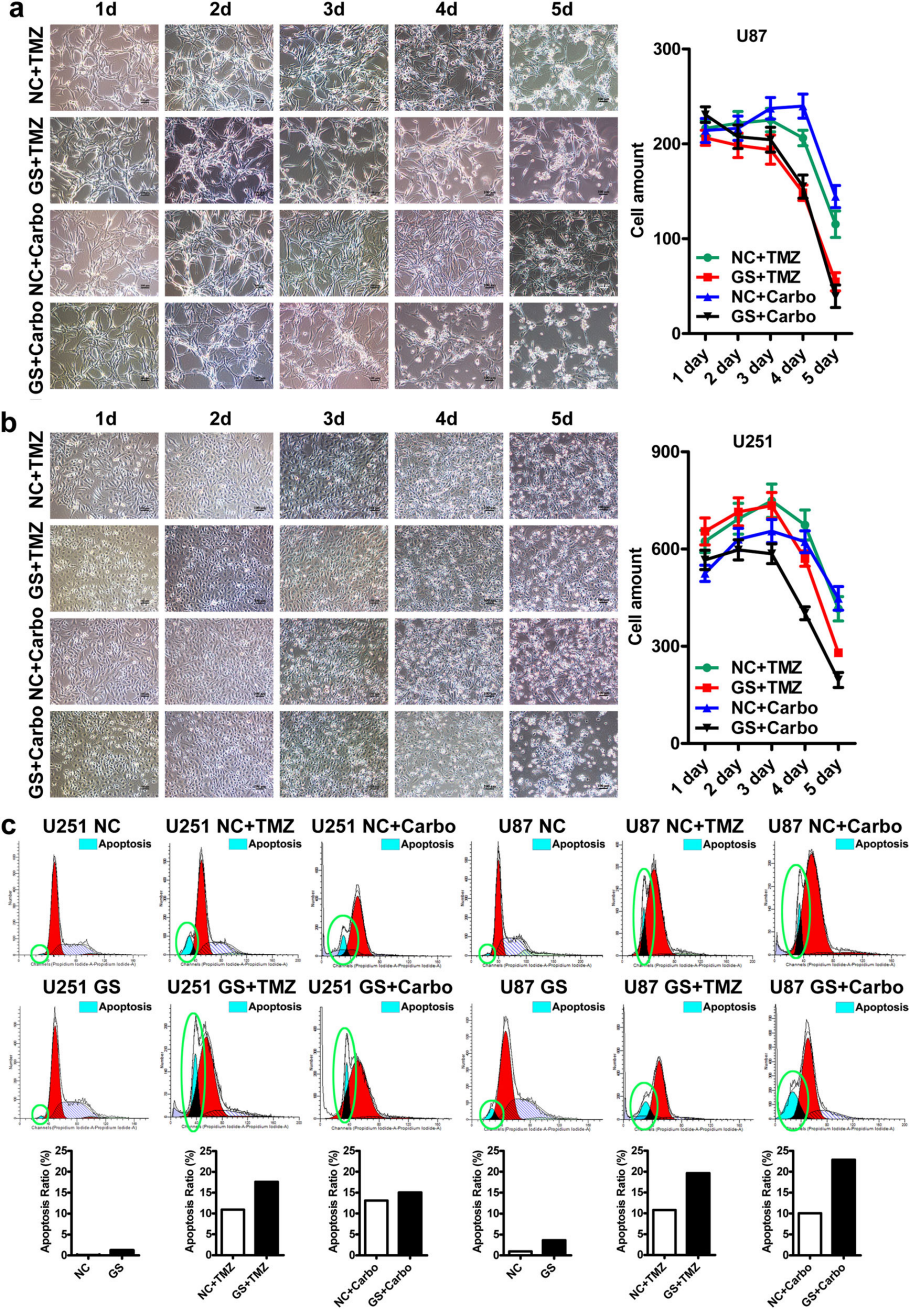
Glucose starvation sensitizes glioblastoma cells to chemotherapies. **a**–**b** Glucose starvation (1.0 g/L) rendered both U87 (**a**) and U251 (**b**) cells more sensitive to chemotherapeutic drugs. By day 5, 40–50% cell death was induced by temozolomide (TMZ, 200 μM) or carboplatin (Carbo, 50 μM) treatment in U87 (**a**) and U251 cells (**b**) under normal glucose condition (4.5 g/L). The cell death rate was nearly doubled to 70–90% under glucose starvation condition. **c** Flow cytometry analysis by PI staining confirmed the synergetic cytotoxic effects between chemotherapies and glucose starvation. Dying cells were identified as the hypodiploids as denoted by the green circles. As depicted, the cell death rates in the lower panels were higher than that of the upper panels. In particular, when chemotherapeutic drugs were combined with glucose starvation as in lower panels (GS + TMZ, GS + Carbo), there were much more hypodiploid cells

It is known that glucose starvation sensitizes cancer cells to chemotherapies. To confirm that, we treated GBM cell lines (U87 and U251) with two widely used chemotherapeutic drugs: temozolomide (TMZ, 200 μM) and carboplatin (Carbo, 50 μM), under normal (4.5 g/L) and low glucose (1.0 g/L) conditions. Glucose starvation rendered both U87 and U251 more sensitive to the two drugs (Fig. 1a, b). The cytotoxic effect was progressive and by day 5, temozolomide or carboplatin treatment caused 40–60% cell loss in U87 and U251 cells. Glucose starvation nearly doubled the cell loss to 70–90%. Although, as revealed by flow cytometry analyses, it alone had marginal cytotoxic effects, the glucose starvation substantially enhanced the cytotoxicity of the two drugs and nearly doubled the cell death rate (10–15% vs 20–25%) with only 2 days of drug treatment, confirming synergetic cytotoxic effects between glucose starvation and drug treatments (Fig. 1c).

### Glucose starvation induces glioblastoma cells to enter quiescence accompanied by enhanced autophagy

**Fig. 2:**
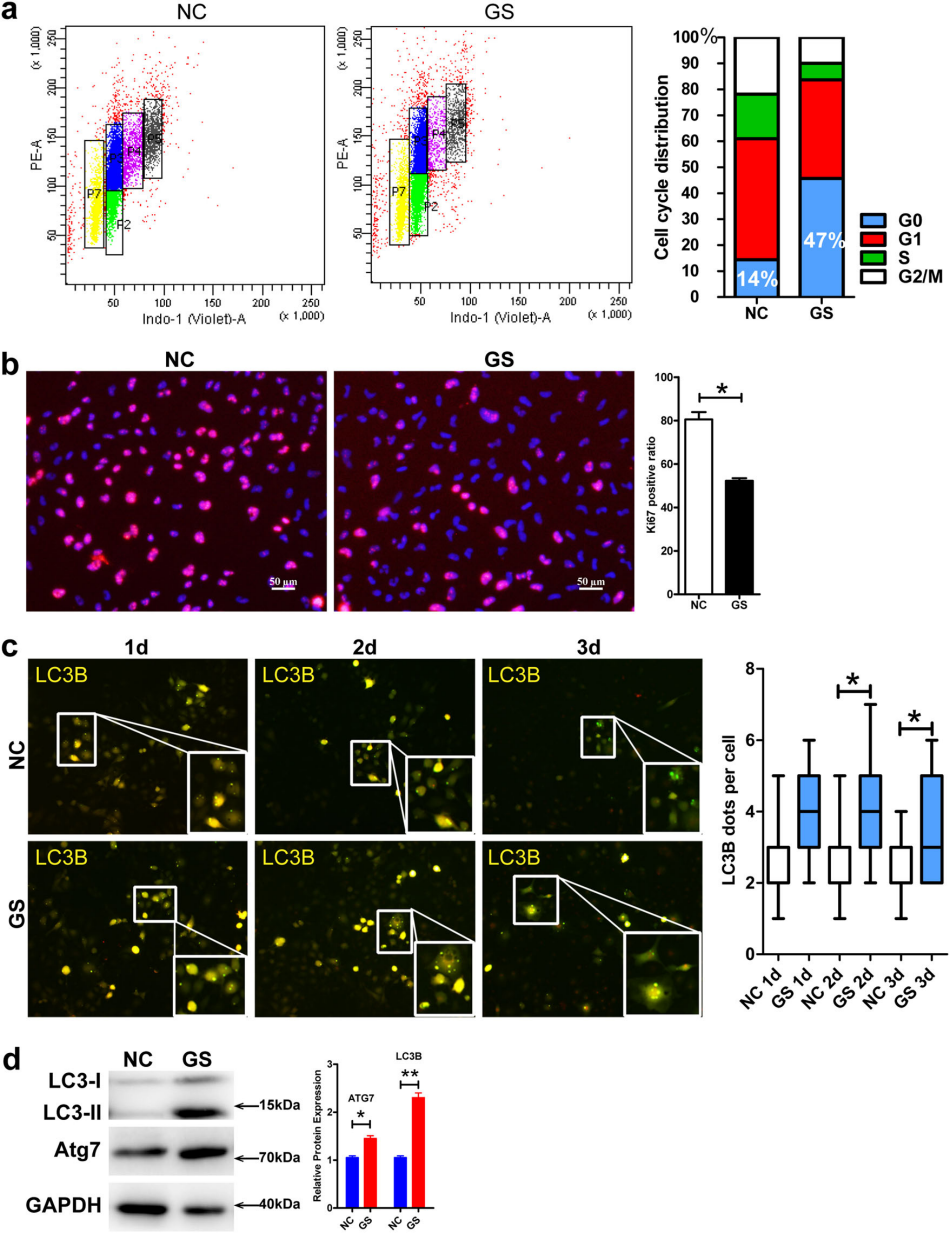
Glucose starvation induces glioblastoma cells to exit cell cycle, enter quiescence, and upregulate autophagy. **a** Under normal glucose condition, more than 80% GBM cells were cycling with only about 15% cells in G0 phase with low RNA content as denoted by PY staining. In contrast, with glucose starvation, only a little over 50% cells were cycling and 47% cells persisted as quiescent cells in G0 phase. **b** Consistent with flow cytometry analysis, there was a 30% decrease in Ki67^+^ proliferating cells with glucose starvation, compared to that with normal condition (*P < 0.05). **c** Glucose starvation upregulates autophagy as determined by the AAV-mRFP-GFP-LC3B reporter. The formation of autophagosomes representing autophagic activity was identified by yellow puncta containing both GFP and RFP signals. Quantification showed that there were significantly more yellow puncta with glucose starvation (lower panels) than that with normal condition (upper panels, starting from the second day, *P < 0.05). **d** Western blot analyses showed that the expression and cleavage of LC3B and the expression of ATG7 were significantly increased with glucose starvation. GAPDH served as an internal control (*P < 0.05, **P < 0.01). NC normal glucose condition, GS glucose starvation condition

Although combination of chemotherapeutic drugs with the glucose starvation induced massive GBM cell death, there were always small subsets of cells that escaped from the treatment and persisted. When closely examined, the flow cytometry analyses above revealed a general absence of G2/M phase in the surviving cells, most of which were in G1 phase (Fig. 1c), suggesting the surviving cells had entered G1 arrest or quiescence-like states. Thus, we speculated that glucose starvation might induce subsets of GBM cells into quiescence, incurring chemoresistance. We set off to determine the cell cycle and metabolic states by flow cytometry analyses of Hoechst 33342 and Pyronin Y, a standard cell cycle analysis method capable to discern G1 and G0 states (Fig. 2a). With 2 days of treatment, while under normal growth condition (4.5 g/L glucose), only 18% GBM cells exited cell cycle, glucose starvation induced 46% to exit cell cycle and enter quiescence. Consistent with the cell cycle analysis, Ki67 staining showed a substantial decrease by 30% (from 80 to 50%) with the glucose starvation (Fig. 2b and Supplementary Figure 1a, *P < 0.05).

Although it can starve and sensitize fast proliferating cells to death stimuli such as chemotherapeutic drugs, glucose starvation also stimulates autophagy, which could drive some starved cells into quiescence and incur chemoresistance. Thus, we wondered whether autophagy was involved in the glucose starvation-induced GBM cell quiescence. We employed the AAV-mRFP-GFP-LC3B reporter and analyzed autophagy under normal and glucose starvation conditions. Twenty-four hours after infection, cells were switched to either normal or glucose starvation condition. The formation of autophagosomes, as indicated by the yellow puncta (emission of both green and red fluorescence), was visibly enhanced with glucose starvation starting from a first day and a second day, respectively, for U87 and U251 cells, nearly doubled at the second day for both the cells (Fig. 2c and Supplementary Figure 1b, **P < 0.01 in U87 cells, *P < 0.05 in U251 cells). Consistent with the reporter analyses, the glucose starvation induced the expression and/or the cleavage of ATG7 and LC3B, two key autophagy molecules and markers, as determined by western blot analyses (Fig. 2d).

### Autophagy promotes glioblastoma cell quiescence, incurring chemoresistance

**Fig. 3:**
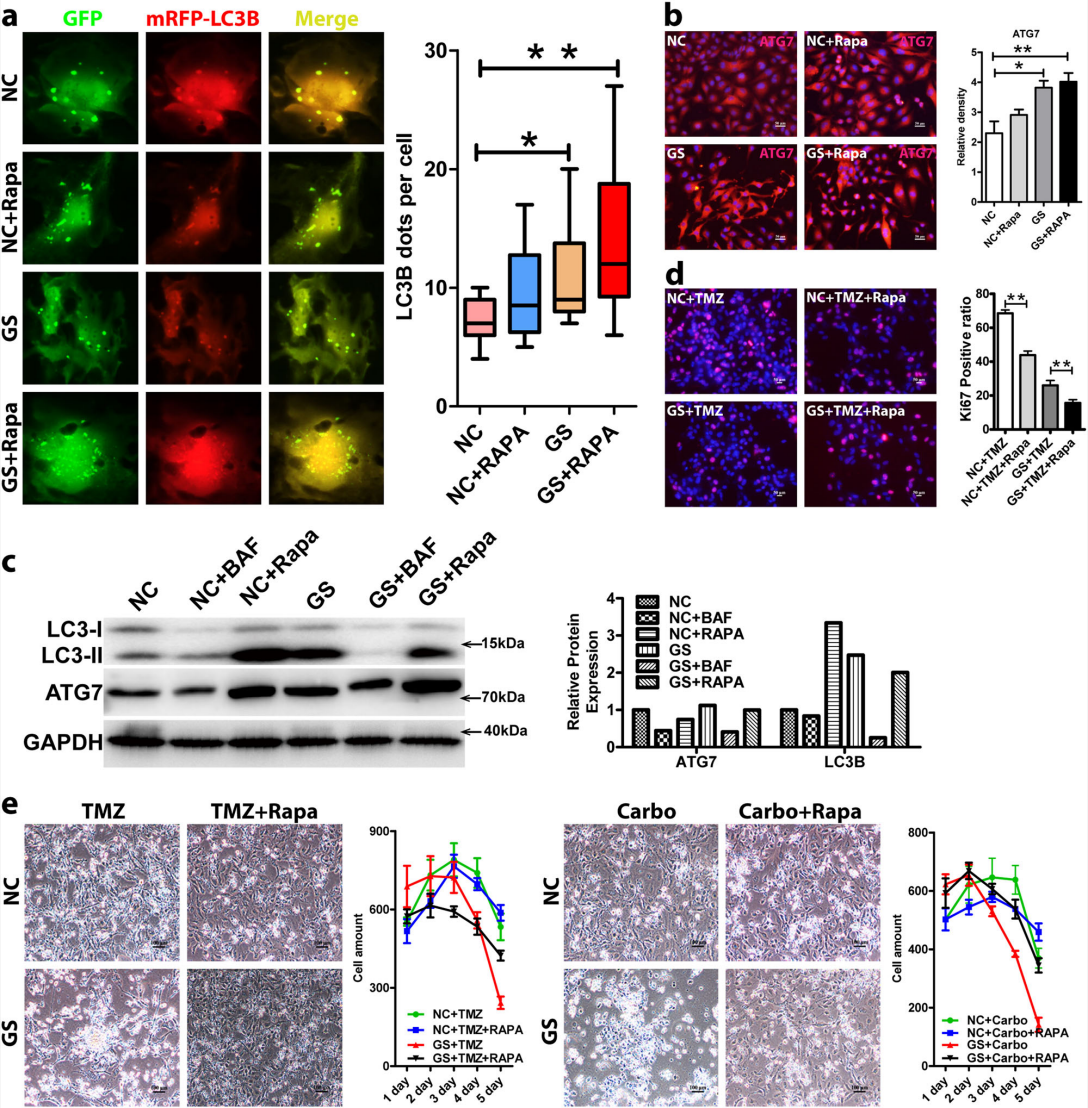
Autophagy promotes glioblastoma cell quiescence, incurring chemoresistance. **a** Rapamycin-enhanced autophagy (lower panels) determined by the AAV-mRFP-GFP-LC3B reporter (*P < 0.05, **P < 0.01). Under normal (upper panels) and glucose starvation condition, there were more yellow puncta with rapamycin than that without the treatment. Combination of rapamycin and glucose starvation induced more formation of autophagosomes (**P < 0.01). In particular, there were significantly more cells of high autophagic activity in the combined treatment group than any of the other treatment ones. **b** Rapamycin induced the expression of ATG7 in GBM cells under normal (upper panels) and glucose starvation condition (lower panels) determined by immunofluorescence staining. **c** Rapamycin induced the expression/cleavage of ATG7 and LC3B under both normal and glucose starvation conditions determined by western blot analysis, confirming rapamycin-inducing autophagy activity. NC normal glucose condition, GS glucose starvation condition, RAPA rapamycin. **d** Ki67 staining showed that GBM cell proliferation was significantly inhibited by 2-day rapamycin treatment under both normal and glucose starvation conditions (**P < 0.01). **e** Rapamycin desensitized glucose-starved GBM cells to chemotherapeutic drugs and promoted their survival. Treatment with either TMZ (200 μM) or Carbo (50 μM) induced over 50 and 80% GBM cell death with normal and glucose starvation conditions, respectively. Although it had little survival effect under normal condition, rapamycin dramatically reduced the cytotoxicity and rescued cell survival under glucose starvation condition. In the combined group, over 50% GBM cells survived, a twofold increase compared to that with the glucose starvation alone group. TMZ temozolomide, Carbo carboplatin

To investigate whether autophagy underlies the chemoresistance of U87 and U251, we subjected the two cell lines to chemotherapeutic drugs under normal and glucose starvation conditions with or without rapamycin, an autophagy agonist. As expected, glucose starvation and rapamycin treatment enhanced autophagic activity indicated by yellow autophagosome puncta (*P < 0.05) (Fig. 3a and Supplementary Figure 2a). Combination of the two induced more formation of autophagosomes (**P < 0.01). In particular, there were significantly more cells of high autophagic activity in the combined treatment group than any of the other treatment. Presumably, it was these cells that have entered quiescence and survived, incurring chemoresistance (Fig. 3a). Consistent with the reporter analyses, the expression of ATG7 was upregulated by rapamycin treatment, determined by immunofluorescence staining and western blot analyses (Fig. 3b, c), and the expression and cleavage of LC3B was also induced determined by western blot analysis (Fig. 3c). As a result, the enhanced autophagy drove cells to quiescence as determined by Ki67 staining (Fig. 3d). Both glucose starvation and rapamycin treatment decreased the proliferation and combining the two had an addictive effect. Treatment with either temozolomide or carboplatin induced about 50 and 80% GBM cell death under normal and glucose starvation conditions, respectively (Fig. 3e and Supplementary Figure 2b). Rapamycin treatment did not considerably rescue cell survival under normal growth condition. In contrast, rapamycin dramatically reduced the cytotoxicity and rescued cell survival under glucose starvation condition. In the combined group, over 50% GBM cells survived, a twofold increase compared to that with the glucose starvation alone group (Fig. 3e and Supplementary Figure 2b). Taken together, these results strongly suggest that autophagy may underlie glucose starvation inducing cancer cell quiescence, survival, and chemoresistance.

### Blocking autophagy alleviates chemoresistance of glioblastoma cells

**Fig. 4:**
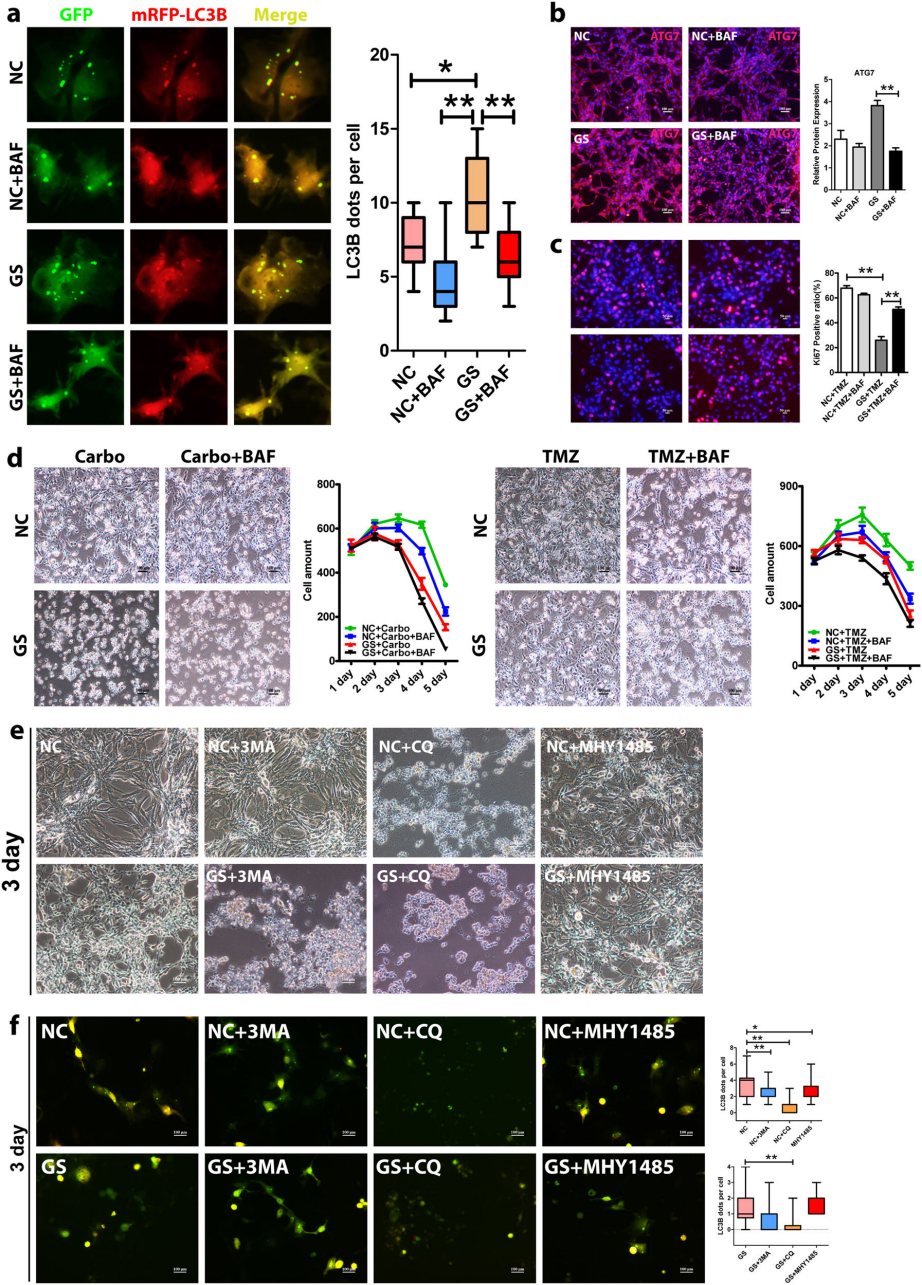
Autophagy inhibition alleviates chemoresistance of glioblastoma cells. **a** Bafilomycin A1 (BAF) inhibited autophagy (lower panels) determined by the AAV-mRFP-GFP-LC3B reporter (*P < 0.05, **P < 0.01). Under normal (upper panels) and glucose starvation condition, there were significantly less yellow puncta with bafilomycin A1 than that without the treatment (*P < 0.05, **P < 0.01). **b** BAF downregulated the expression of ATG7 only slightly with normal (upper panels) but significantly with glucose starvation condition (lower panels) as examined by immunofluorescence staining. In addition, as shown in Fig. 3c, bafilomycin A1 repressed the expression/cleavage of ATG7 and LC3B under both normal and glucose starvation conditions determined by western blot analysis. NC normal glucose condition, GS glucose starvation condition. **c** Ki67 staining showed that GBM cell proliferation was significantly enhanced by bafilomycin A1 under glucose starvation conditions (**P < 0.01). After 2-day treatment of BAF, the percentage of Ki67-positive cells nearly doubled (26–50%) under glucose starvation condition (*P < 0.05, **P < 0.01). **d** BAF further sensitized glucose-starved GBM cells to chemotherapeutic drugs. The BAF enhanced the cytotoxicity under both normal and glucose starvation conditions. In particular, it effectively killed the subsets of cells that otherwise would have had entered quiescence, escaping from the chemotherapeutic drugs under the glucose starvation condition. As well, autophagy inhibition rendered the cells die faster and earlier (also refer to Supplementary Figure 3). **e** The synergistic effect of autophagy inhibition with chemotherapeutic drugs was independently confirmed by two other autophagy inhibitors of 3-MA and CQ. Nonetheless, the autophagy inhibitor MHY1485 failed to produce synergistic effect. 3-MA, 3-methyladenine, CQ hydroxychloroquine sulfate. **f** As revealed by the AAV-mRFP-GFP-LC3B reporter, both 3-MA and CQ effectively inhibited autophagy but MHY1485 failed to significantly repress autophagy, explaining its failure to produce synergistic effect

Next, we sought to examine whether autophagy was required for the chemoresistance and if so, whether autophagy inhibition could prevent the acquisition of chemoresistance in GBM cells. Toward that, we treated the GBM cells with chemotherapeutic drugs under normal and glucose starvation conditions with or without bafilomycin A1, a specific autophagy inhibitor (lysosomal acidification inhibition). As expected, bafilomycin A1 effectively inhibited autophagy activity in all experimental conditions, determined, respectively, by the AAV-mRFP-GFP-LC3B reporter (Fig. 4a and Supplementary Figure 3a). Consistent with the reporter analyses, the expression of ATG7 was repressed by bafilomycin A1 determined by immunofluorescence staining and western blot analyses (Figs. 3c and 4b), and the expression and cleavage of LC3B was also repressed as determined by western blot analysis (Fig. 3c). Importantly, bafilomycin A1 prevented cancer cells entering quiescence. Under glucose starvation condition, addition of bafilomycin A1 increased cell proliferation by twofold (Fig. 4c). As such, autophagy inhibition under the glucose starvation condition sensitized GBM cells to cytotoxic chemotherapies. The synergistic effects between bafilomycin A1 and chemotherapies essentially eradicated the subsets of cells that otherwise would have had exited cell cycle and persisted in quiescence-like states, acquiring chemoresistance upon glucose starvation (Fig. 4d and Supplementary Figure 3b). Since bafilomycin A1 could have non-autophagy-related activities, we performed confirmatory experiments with additional autophagy inhibitors including hydroxychloroquine sulfate (lysosomal acidification inhibitor), 3-methyladenine (3-MA, PI3K inhibitor), and MHY1485 (mTOR agonist). Similar to bafilomycin A1, both hydroxychloroquine sulfate and 3-MA had synergistic cytotoxic effect with chemotherapies. They not only sensitized the cells to death, but also substantially sped up the death. Nonetheless, to our surprise, MHY1485 failed to produce an effect. To address this puzzling observation, we utilized the AAV-mRFP-GFP-LC3B reporter and examined the autophagic activity upon the treatment of each inhibitor. Similar to bafilomycin A1, both hydroxychloroquine sulfate and 3-MA successfully inhibited autophagy. In contrast, MHY1485 failed to effectively inhibit autophagy, explaining its failure to have synergistic effect with chemotherapies.

### Identification of candidate genes and pathways regulated by autophagy in glioblastoma cells

**Fig. 5:**
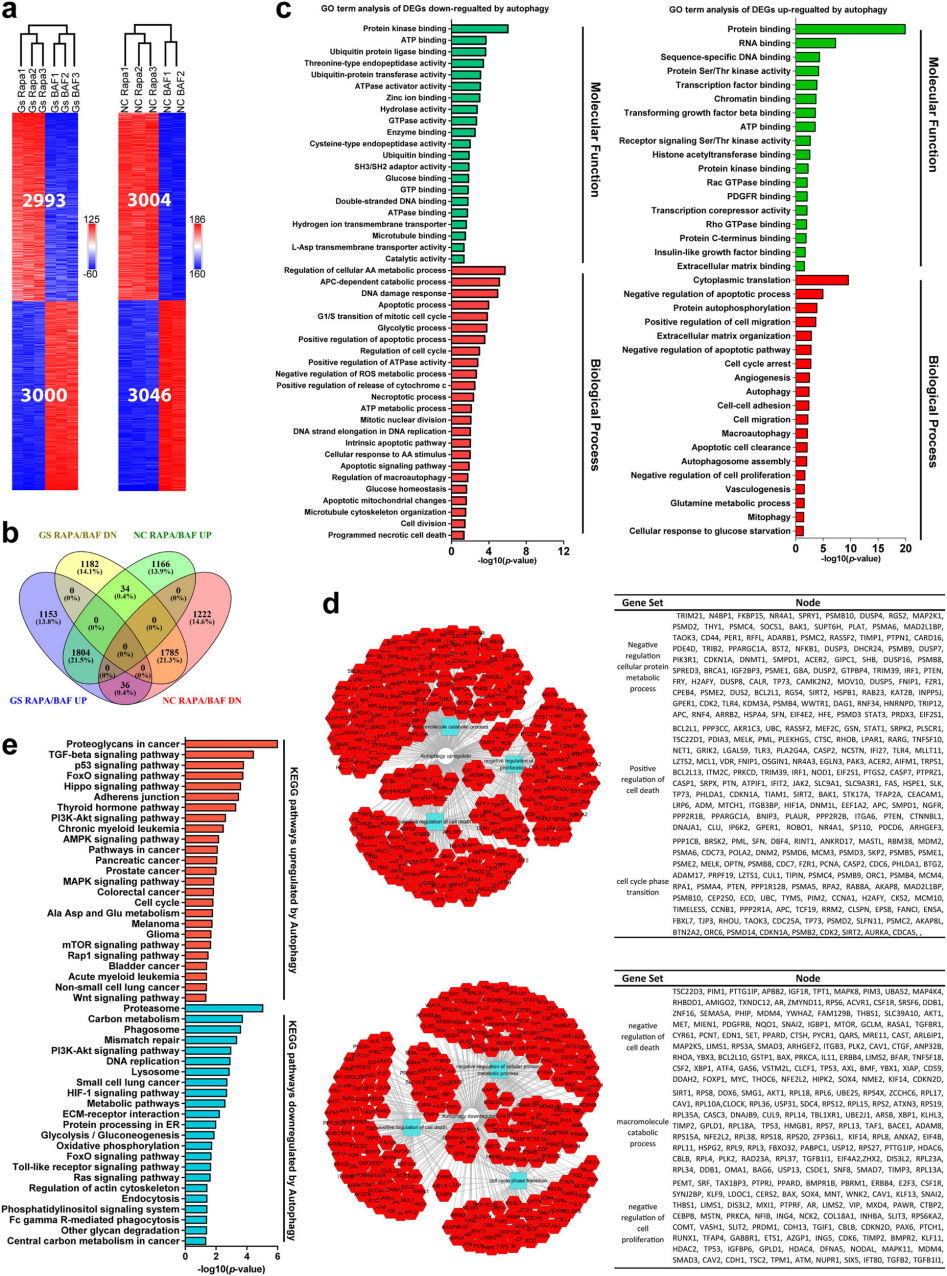
Key genes and pathways regulated by autophagy in glioblastoma. **a** Clustering analyses revealed differentially expressed genes (DEGs) between the experimental groups with different autophagy manipulations. Under normal condition, there were 3004 genes upregulated and 3046 genes downregulated by autophagy. Under glucose starvation condition, there were 2993 genes upregulated and 3000 genes downregulated by autophagy. **b** A total of 1804 genes were upregulated and 1785 were downregulated significantly by autophagy under both normal and glucose starvation conditions. **c** Gene ontology analysis of autophagy-regulated genes revealed many important processes related to cell metabolism/autophagy, cell cycle, death and survival etc., consistent with the phenotypical changes. **d** PPI network analysis revealed that the most upregulated modules were related to macromolecule catabolic process, negative regulation of cell proliferation and negative regulation of cell death and the downregulated modules consisted of those related to negative regulation of cellular protein metabolic process, positive regulation of cell death and cell cycle phase transition. **e** KEGG pathway analysis revealed that DEGs altered by autophagy were enriched in cell metabolism, DNA replication, cell growth, and cell cycle as well as cancer-related pathways

To elucidate autophagy-mediated chemoresistance, we performed transcriptomic profiling by RNA-Seq. Since autophagy manipulation was used to prime the cells (becoming either more resistant or more sensitive to chemotherapies) and might only influence small subsets of cancer cells of stem cell-like properties, we did not expect there would be a dramatic expression change with most of the genes from population analysis of heterogeneous cells. Therefore, we designed the experiments and the analyses to look for consistent changes across different replicates and different experimental conditions. We collected RNA samples from GBM cells cultured with rapamycin or bafilomycin A1 under normal or glucose starvation condition, respectively. Pairwise comparisons between rapamycin and bafilomycin A1 treatment groups should reflect the effect of autophagy. For glucose starvation condition, there were 5993 differentially expressed genes (DEGs) and for normal glucose condition, there were 6050 DEGs (Fig. 5a). Among them, 1804 were upregulated and 1785 were downregulated by autophagy in both normal and glucose starvation conditions (Fig. 5b).

The DEGs upregulated by autophagy were mostly associated with glucose starvation response, autophagy, cell cycle arrest, negative regulation of apoptosis, enriched in cellular components of mitochondrial, endoplasmic reticulum, autophagosome, and extracellular matrix (Fig. 5c and Supplementary Table 2). The DEGs downregulated by autophagy were associated with positive regulation of cell cycle, microtubule, cytoskeleton organization, apoptotic signaling pathway, negative regulation of reactive oxygen species (ROS) metabolic process, and regulation of cellular amino acid metabolic process, mainly located in microtubule cytoskeleton, centrosome, mitochondrial, and proteasome complex (Fig. 5c and Supplementary Table 1).

Further protein–protein interaction (PPI) network analysis produced a number of interesting modules altered by autophagy. The most upregulated modules had to do with macromolecule catabolic process, negative regulation of cell proliferation, and negative regulation of cell death. The most downregulated modules consisted of those related to negative regulation of cellular protein metabolic process, positive regulation of cell death, and cell cycle phase transition. These results are in line with the role of autophagy promoting GBM cell quiescence and survival.

KEGG pathway analysis revealed that DEGs upregulated by autophagy were enriched in tumor-related pathways such as proteoglycans in cancer, p53, FoxO, PI3K-AKT, AMPK, Hippo signaling, cancer, and cell cycle. The downregulated DEGs were mainly related with proteasome, carbon metabolism, DNA replication, FoxO, PI3K-AKT and HIF-1 signaling, metabolic pathways, extracellular matrix–receptor interaction, oxidative phosphorylation, and central carbon metabolism in cancer (Fig. 5d and Supplementary Table 3). Taken together, these results suggest that autophagy regulates cancer cell metabolism, cell cycle and apoptosis, induces quiescence, supports survival and confer cytotoxic resistance, at least partially explaining autophagy-mediated chemoresistance.

**Fig. 6:**
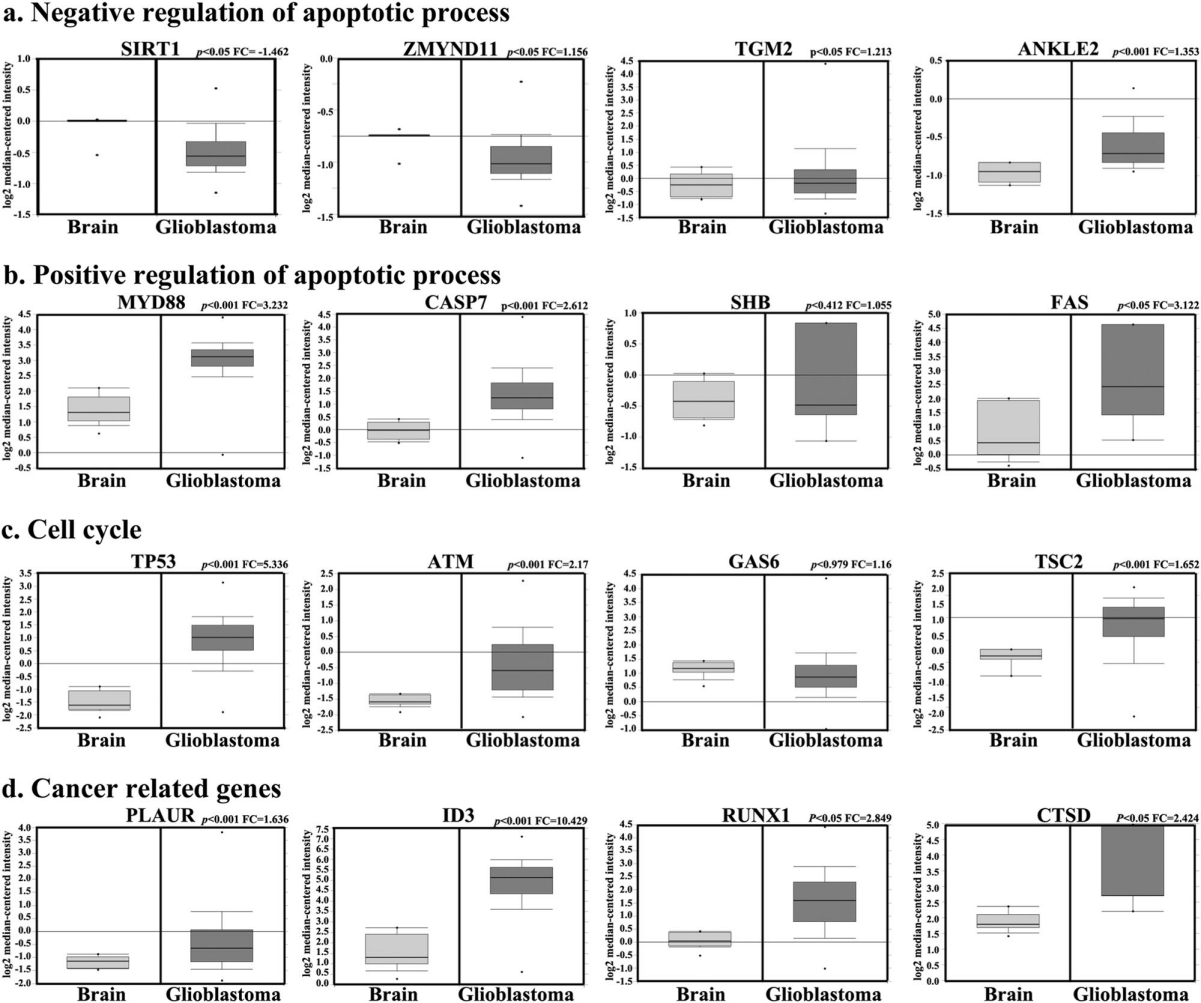
Autophagy-regulated genes differentially expressed between glioblastoma and normal tissues as revealed by TCGA data mining with Oncomine platform. A high correlation between the TCGA database and our results was evident. A large number of the DEGs from our RNA-seq analysis were differentially expressed between GBM and normal tissues. Representative examples were grouped and presented based on their biological functions **a** negative regulation of apoptotic process; **b** positive regulation of apoptotic process; **c** cell cycle; **d** cancer-related gene. Most of these genes were well-known molecules associated with cancers while the rest may merit further investigation. One of the genes, *Gas6*, was differentially regulated in GBM cell lines by autophagy but did not show a significant change between GBM and normal tissues because its expression was highly variable among the GBM patient tissues. However, the patients with high expression of *Gas6* had significantly shorter survival time than those with low expression (refer to Fig. 7)

**Fig. 7:**
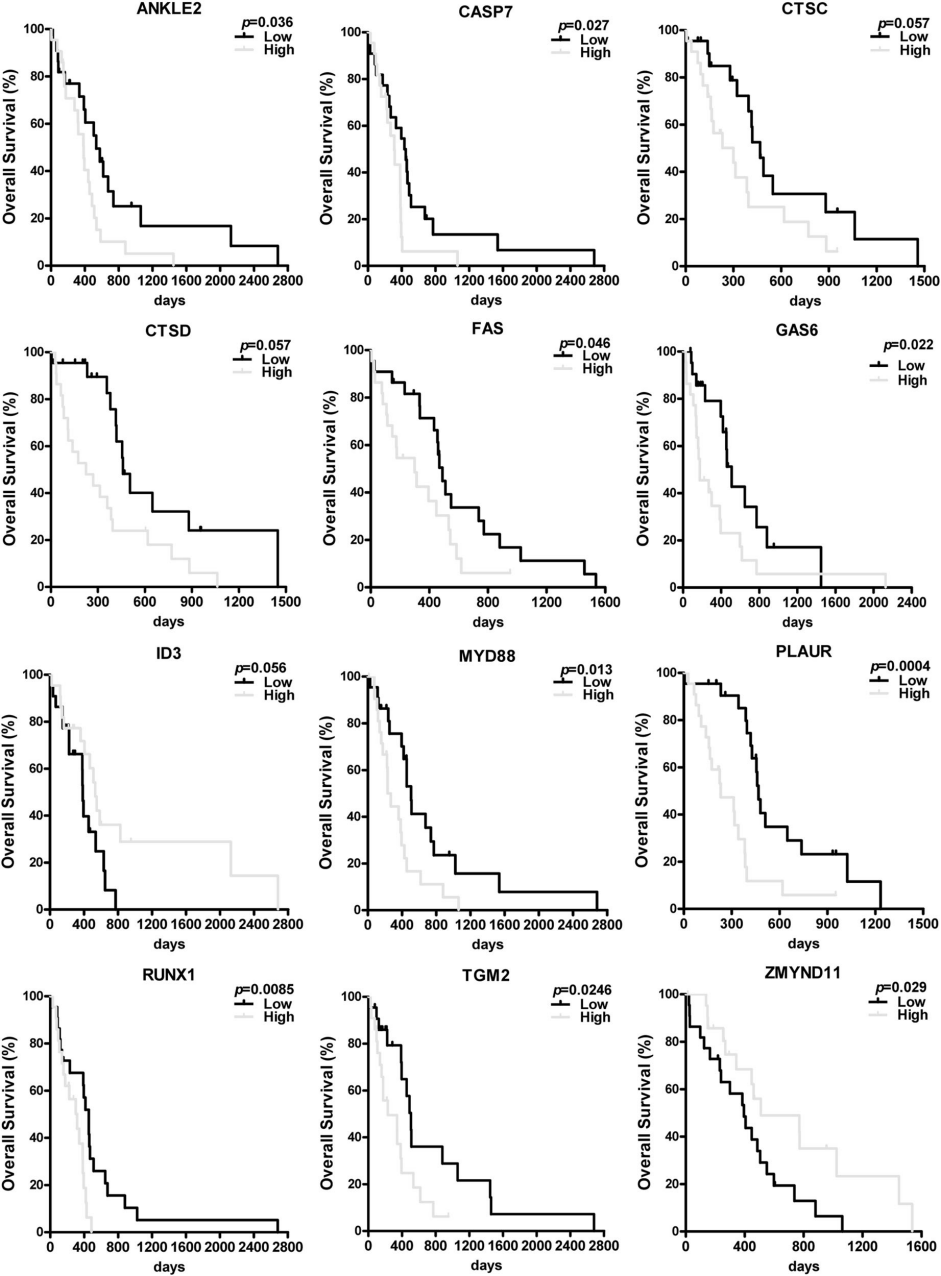
Many differentially expressed genes demonstrated strong prognostic value for glioblastoma patients. Many of the differentially expressed genes demonstrated strong prognostic value for patient survivals. Some of the genes are known associated with tumorigenesis (e.g., *Fas* and *Id3*) while others (e.g., *Ankle2*, *Zmynd11*, and *Tgm2*) have not been previously reported in cancers and/or GBM. Nonetheless, the known functions of these genes are highly suggestive for their potential roles in brain tumors. For each DEG, the 152 patients were arbitrarily divided as 76 patients with high expression versus 76 patients with low expression

Although our in vitro phenotypic studies and transcriptomic profiling supported a role of autophagy in GBM chemoresistance, it remained to be determined whether these insights had any clinical relevance. Hence, we took advantage of the TCGA database and obtained expression data of the interested pathways and genes. A high correlation between the TCGA database and our results was evident. Out of the 114 DEGs related to positive regulation of apoptosis, 52 were differentially expressed between GBM and normal tissues with 42 upregulated and 10 downregulated. Out of the 77 DEGs related to negative regulation of apoptosis, 49 were altered. Out of the 35 DEGs related to autophagy, 17 were changed. Out of the 54 DEGs related to cancer, 23 were altered. Out of the 40 DEGs related to cell cycle arrest, 20 were altered. Out of the 59 DEGs related to cell cycle, 35 were altered. Many of these genes are well-known molecules involved tumorigenesis with some novel ones worth further investigation (Fig. 6 and Supplementary Table 4). In addition, one of the genes we included in Fig. 6, *Gas6* was differentially regulated in GBM cell lines by autophagy but did not show a significant change between GBM and normal tissues. When examined in details, the expression of the gene was highly variable among the GBM patient tissues. But as it turns out, the patients with high expression of *Gas6* had significantly shorter survival time than those with low expression (Fig. 7).

Importantly, in each of these pathways, we found that the expression of many genes not only correlated with GBM progression, but also had strong effects on patient survival time, suggesting their biological significance in GBM. High expression of anti-apoptotic genes, e.g., *Ankle2, Tgm2*, and *Myd88*, negatively impacted patient survival but that of *Zmynd11* manifested a positive effect (Fig. 7). Similarly, low expression of cell cycle genes, e.g., *Fas*,* Casp7*, *Ctsd*, and *Gas6*, significantly correlated with a longer survival time. We also included some genes from other pathways that affected patient survival time, such as *Runx1* (pathway in cancer), *Id3* (amino acid stimulus) and, *Plaur* (cytochrome *c* production).

### The state of autophagy dictates the sensitivity of GBM primary cells to chemotherapy

**Fig. 8:**
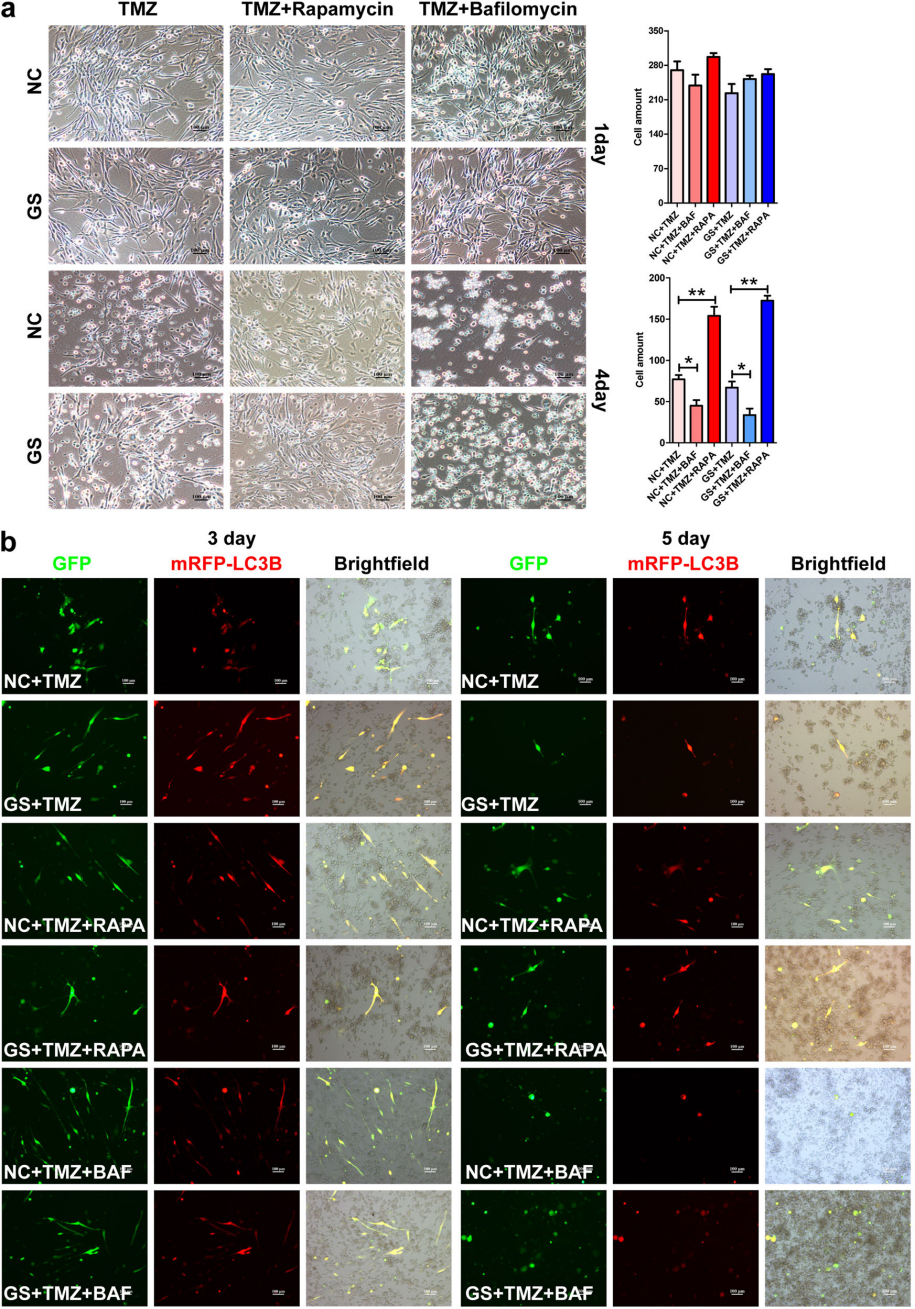
Autophagy modulates the sensitivity of GBM primary cells to chemotherapy. **a** The effects of autophagy to chemoresistance were confirmed with primary cells from GBM patients. Similar to what was observed with GBM cell lines, upon 5-day drug treatment, while autophagy inhibition by bafilomycin A1 sensitized the primary cancer cells to death, autophagy induction by rapamycin dramatically attenuated the cytotoxicity of chemotherapy and promoted cancer cell survival. **b** As determined by the AAV-mRFP-GFP-LC3B reporter assay, for all the groups, most of the surviving cells manifested high level of autophagic activity after 5-day treatment, suggesting that autophagy underlies the chemoresistance of GBM primary cells

Finally, we wished to determine whether the state of autophagy influences the chemo-sensitivity of GBM primary cells. Similar to what were observed with U87 and U251, at the first day, no obvious difference was observed between the groups. But, 4-day treatment of bafilomycin A1 (autophagy inhibitor) dramatically increased the cytotoxicity of chemotherapies. The GBM primary cells underwent massive cell death regardless of the glucose states (Fig. 8a). In contrast, 4 days of rapamycin treatment rendered chemotherapies ineffective and a high percentage of the cells survived and became resistant to the drugs (Fig. 8a). Importantly, by the end of the 5-day treatment, for all the groups, most of the surviving cells manifested high level of autophagic activity (Fig. 8b). Taken together, these results suggest that the state of autophagy dictates the chemo-sensitivity of GBM primary cells, at least partially responsible for drug resistance.

## Discussion

Metabolic reprogramming is vital for cancer cell survival and progression. Metabolism-oriented therapies target mainly the fast proliferating “bulk tumor” cells. But the slow dividing and/or dormant cancer stem cells may escape the cytotoxicity, leading to drug resistance and cancer relapse despite an initial response. Thus, it is of paramount importance to elucidate mechanisms promoting cancer cell quiescence and survival upon dietary restriction and chemotherapy. Our works suggest that although upon glucose starvation, the majority of cancer cells succumb to chemotherapeutic cytotoxicity, subsets of cancer cells can upregulate their autophagic activity and enter quiescence, acquiring survival advantage and ultimately chemoresistance. Autophagy inhibition effectively prevented cancer cells entering quiescence and restored their sensitivity to chemotherapies. Further transcriptomic analysis and TCGA data mining revealed that autophagy could modulate a range of clinically important pathways and genes, and coordinate cell metabolic, cell cycle, and apoptotic activities, at least partially explaining autophagy-mediated chemoresistance.

Results from both animal studies and clinical trials suggest that dietary restriction is of important tumor therapeutic value^27,28^. A prominent example from the Zhang group reported a remarkable inhibitory effect of fasting on the progression of acute lymphoblastic leukemia^29^. The ketogenic diet of high fat, moderate protein, and very low carbohydrate evokes a physiological state similar to what exercise or fasting does, justifying its possible use in malignancy treatment. Although proofs for clinical efficacy remain limited, human population and animal model studies both supported that dietary therapy has potential to improve outcome for patients with GBM and other malignant brain cancers^27^. An obvious synergistic effect between glucose starvation and chemotherapies as observed in our study supports the notion that dietary restriction can sensitize GBM cells to chemotherapy.

Although dietary restriction displayed potentials in cancer treatment, drug resistance and disease relapse are highly expected. Tumors are constantly evolving and cancer cells reprogram their metabolism under therapeutic stress. Upon combined metabolic starvation and chemotherapy, the majority of the fast proliferating cells undergo death. But subsets of stem cell-like cancer cells may exit cell cycle and stay quiescent or dormant in an acidic microenvironment lack of sufficient oxygen and nutrients. These cells can evade treatment and become culprit for relapse. A major pathway responsive to dietary change is autophagy, which is associated with tumor development and drug resistance. But the role of autophagy in cancer and cancer treatment is complex. The notion of metabolic starvation is partially rooted in excessive autophagy driving self-eating and cell death, and enhancing chemotherapeutic efficacy^17^. Deficiency in autophagy as in the *Pten* and *Beclin1* heterozygous knockouts resulted in tumorigenesis^30,31^. It is also supported by clinical data that 40–75% of ovarian and prostate cancers with *Beclin1* deficiency were related to aggressive phenotypes^32^. On the other hand, cumulative evidence indicates that autophagy mostly leads to cancer cell survival and resistance to treatment. As a matter of fact, in Drosophila melanogaster model, early stage tumor growth and invasion is dependent on autophagy and autophagy inhibitors restrain tumor progression^11,33^. Taken together, the role of autophagy in tumor initiation and drug resistance is likely context specific.

Our present work attests the complexity. Glucose starvation enhanced autophagic activity and manifested strong synergistic cytotoxic effect with chemotherapy. Nonetheless, a small subset of GBM cells with active autophagy underwent cell cycle arrest and adopted a quiescence-like state, resistant to cytotoxicity and persisted as potential culprits for relapse. These results are in line with earlier studies^33–36^. Although short-term starvation seemed to boost the sensitivity of chemotherapy and radiotherapy, and prolonged survival time, more than 80% patients died from GBM progression^33^. Importantly, rapamycin treatment significantly decreased the cytotoxicity of chemotherapeutic drugs with over 50% GBM cells survived in both normal and glucose starvation conditions. In direct contrast, bafilomycin A1 treatment blocked autophagy and essentially eradicated all tumor cells when combined with glucose starvation and chemotherapeutic drugs.

The mechanisms underlying the roles of autophagy in cancer and cancer treatment remain poorly defined^28, 37,38^. What is increasingly clear is that the mechanisms are likely complex, dynamic, and context-dependent. Diverse pathways and processes can come into play including autophagy-mediated effects on cell death, cell cycle arrest, tumor promoting inflammation, tumor microenvironments, immunogenicity, immune cell cytotoxicity, and checkpoints^28, 37,38^. In the present study, we hoped to utilize in vitro modeling systems to elucidate the cell autonomous mechanisms in an unbiased manner through combining RNA-seq analysis with TCGA data mining. As with phenotypical changes, autophagy manipulation profoundly impacted the molecular activity of GBM cells. Numerous processes underwent dramatic changes, many associated with major pathways and processes controlling either cell identity (transcriptional control and RNA splicing, chromatin modification, etc.), metabolism (autophagy, AMPK signaling, ATG/ULS complex, Asp and Glut metabolism, cell organelles), or cell fate (ECM, DNA damage, apoptosis, necrosis, cell cycle, etc.). Consistent with the phenotypical changes, enhancing autophagy downregulated genes related to autophagy, cell cycle, apoptosis and anabolic metabolism, and upregulated genes related to catabolic metabolism, cell cycle arrest and survival. In contrast, autophagy inhibition had opposite effect.

Importantly, many of the pathways and genes are known for their roles in control of autophagy, cell cycle, tumorigenesis, and/or cell death. A high percentage of the genes turned out to be key GBM oncogenes or suppressors (e.g.,*Tp53*, *Atm*, *Pten*, *Brca1*, *Tsc2*, *Id3*, *Akt1*, and *Avcr1*), attesting the validity of our model and the power of our analyses. When the results from our RNA-seq analysis were compared with TCGA data, a striking high correlation was evident. Among the genes whose expression has a strong predictive value, we not only identified a number of well-known GBM causing genes (e.g., *Tp53, Atm*, and *Id3*) but also genes that have not been previously implicated in GBM. Among them, ZYMND11 is a H3.3-specific reader of H3.3K36Me3 and has recently been reported as a tumor repressor in breast cancer^39-46^. Its mutations/translocations have also been newly associated with leukemia, developmental delay, and mental retardation. ANKLE2 is a poorly studied nuclear envelope assembly protein with its mutations associated with microcephaly^47–49^. CTSD is a lysosome enzyme involved in autophagy and apoptosis. It has been associated with brain pathology but has not yet been implicated in brain tumor^50–53^. Transglutaminase 2 (TGM2) is a posttranslationally modifying enzyme catalyzing the formation of intermolecular isopeptide bonds between glutamine and lysine side chains. Scattered evidence suggests that TGM2 might be involved in several types of cancers including glioma^54–56^. The known functions of these genes are highly suggestive, highlighting the revealing power of our model and analysis. It would be of high interest to investigate these genes for their roles in GBM tumorigenesis, progression, and/or drug resistance.

## Materials and methods

### Cell culture and glucose starvation

GBM cell lines U87 and U251 were maintained in Dulbecco’s-modified Eagle’s medium (DMEM 4.5 g/L, 11965-092, GIBCO) supplemented with 10% fetal bovine serum (FBS, 10099-141, GIBCO). Glucose starvation was introduced by culturing cells in low glucose DMEM (1.0 g/L, 10567-014, GIBCO) supplemented with 10% FBS and the medium was changed every other day. Tissue samples from GBM patients were obtained following the protocol approved by Tongji University School of Medicine and the affiliated hospital. GBM primary cells were derived as previously reported^57^.

### Drug treatment of glioblastoma cells and cell quantification

U87 and U251 cells were seeded in 24-well plates at a density of 10,000 cells/well in normal medium (4.5 g/L glucose) supplemented with 10% FBS. After overnight incubation, cells were subjected to drug treatment for up to 5 days as indicated under normal or glucose starvation medium: temozolomide (200 μM, M2129, Abmole), carboplatin (50 μM, M2288, Abmole), respectively, with or without autophagy inhibitors/agonist, bafilomycin A1 (10 nM, A601116, Sangon), 3-methyladenine (2 mM, S2767, Selleck), hydroxychloroquine (100 nM, S4430, Selleck), MHY1485 (25 μM, S7811, Selleck) and rapamycin (5 μM, S1039, Selleck). Six-phase contrast images per well were taken randomly on each day and the quantification was done by counting the cells present in the field, with six fields each replicate and three replicates each treatment, in total at least 500–3000 cells counted (*P < 0.05, **P < 0.01).

### Immunofluorescence staining

Cells were fixed by 4% paraformaldehyde in PBS for 15 min and permeabilized with 0.3% Triton X-100 for 15 min at room temperature. After 30-min blocking with 3% BSA, cells were incubated with primary antibodies overnight at 4 °C and on a second day, stained with secondary antibodies. The primary antibodies include rabbit anti-Ki67 (1:1000, RM-9106-R7, Thermo) and anti-ATG7 (1:1000, A7360, Abclonal). DAPI was used as counter staining for nuclei. The intensity of the ATG7 staining was semi-quantified by ImageJ.

### Western blotting

Total cell lysates were prepared in RIPA buffer with western blotting performed as previously described^58^. GAPDH was used as a loading control. The primary antibodies used are rabbit anti-ATG7 (1:1000, A7360, Abclonal), anti-LC3B (1:1000, 2775S, Cell Signaling), and mouse anti-GAPDH (1:1000, A10868, Abclonal).

### Cell cycle and cell death analysis

Single cell resuspensions were fixed in pre-cooled 70% ethanol and when needed stored at −20 °C. For cell cycle analysis, fixed cells were first incubated in Hoechst 33342 solution (for DNA content) in 500 μl (0.25 μg/mL) for 15 min at room temperature and then followed by 5-min staining with pyronin (for RNA content) at 0.5 μg/mL on ice before flow cytometry analysis, 10,000 events collected per sample with FACSVerse (BD Biosciences). For cell death analysis, fixed cells were stained by propidium iodide (PI) solution at 50 mg/L on ice for 30 min, following a standard protocol^59^. Dying cells were identified as the hypodiploid with DNA content quantified by PI staining, 10,000 events per sample.

### AAV-mRFP-GFP-LC3B infection

Cells in 24-well plates were infected with AAV-mRFP-GFP-LC3B (MOI 300, HB-AP2100001, Hanbio) according to the manufacturer’s instruction. Twenty-four hours post infection, cells were treated with drugs as described above. Six fluorescence images per well were captured with microscope (Nikon, ECLIPSE Ti) on each day. Green and red fluorescence represented phagophore and autolysosome, respectively, and yellow fluorescence (puncta) represented autophagosome. The autophagic activity generally indicated by formation of autophagosome was determined by quantifying the yellow puncta, six fields per replicate and three replicates per treatment with at least 20–50 cells counted.

### RNA-sequencing analysis

Cells in 6-well plates were harvested after 3-day treatment with total RNA extracted using TRIzol reagent (15596018, Invitrogen). mRNA was enriched with poly-A selection and 50 base pair paired-end RNA-seq was completed on BGISEQ-500 platform at Beijing Genomics Institute (BGI-Shenzhen). Raw reads were filtered using SOAP and SOAPnuke^60^ and clean reads were mapped to transcriptome of RefSeq database using Bowtie2^61^. Gene expression was counted by RSEM^62^, and normalized as TPM (transcripts per kilobase of exon model per million mapped reads). We used DESeq2 to evaluate differential expression and differentially expressed genes were identified by Benjamini and Hochberg-adjusted *P* value (<0.05)^63^. Gene ontology (GO) and KEGG pathway enrichment were analyzed using DAVID^64^.

### TCGA data comparison with Oncomine^TM^ and survival time analysis with Oncolnc

To further validate our results, we subjected the DEGs from our analyses to query the TCGA database through Oncomine^TM^ platform (Thermo. Fisher, Ann Arbor, MI; http://www.oncomine.org) by setting up the following parameters: “Cancer vs Normal analysis” and “Glioblastoma” options (547 GBM patient brain tissues versus 10 normal brain tissues). For survival analysis, Oncolnc platform was used to extract the prognostic value of DEGs on the survival time of 152 glioblastoma patients from TCGA database^65^. For each DEG, the 152 patients were arbitrarily divided as 76 patients with high expression versus 76 patients with low expression according to their expression level of the DEGs.

### Statistical analyses

Unless specified, all data were statistically analyzed in GraphPad Prism Version 5 with significance determined by Student’s *t* test; *P < 0.05, **P < 0.01.

## Electronic supplementary material

Supplementary Information
