# Supplementary material for: Autophagy mediates glucose starvation-induced glioblastoma cell quiescence and chemoresistance through coordinating cell metabolism, cell cycle, and survival
Source: Cell Death Dis. 2018 Feb 12;9(2):213. doi: 10.1038/s41419-017-0242-x (PMC5833690; doi:10.1038/s41419-017-0242-x)
Supplement: Supplementary file 1 — Supplementary Information [file 41419_2017_242_MOESM1_ESM.docx]

**
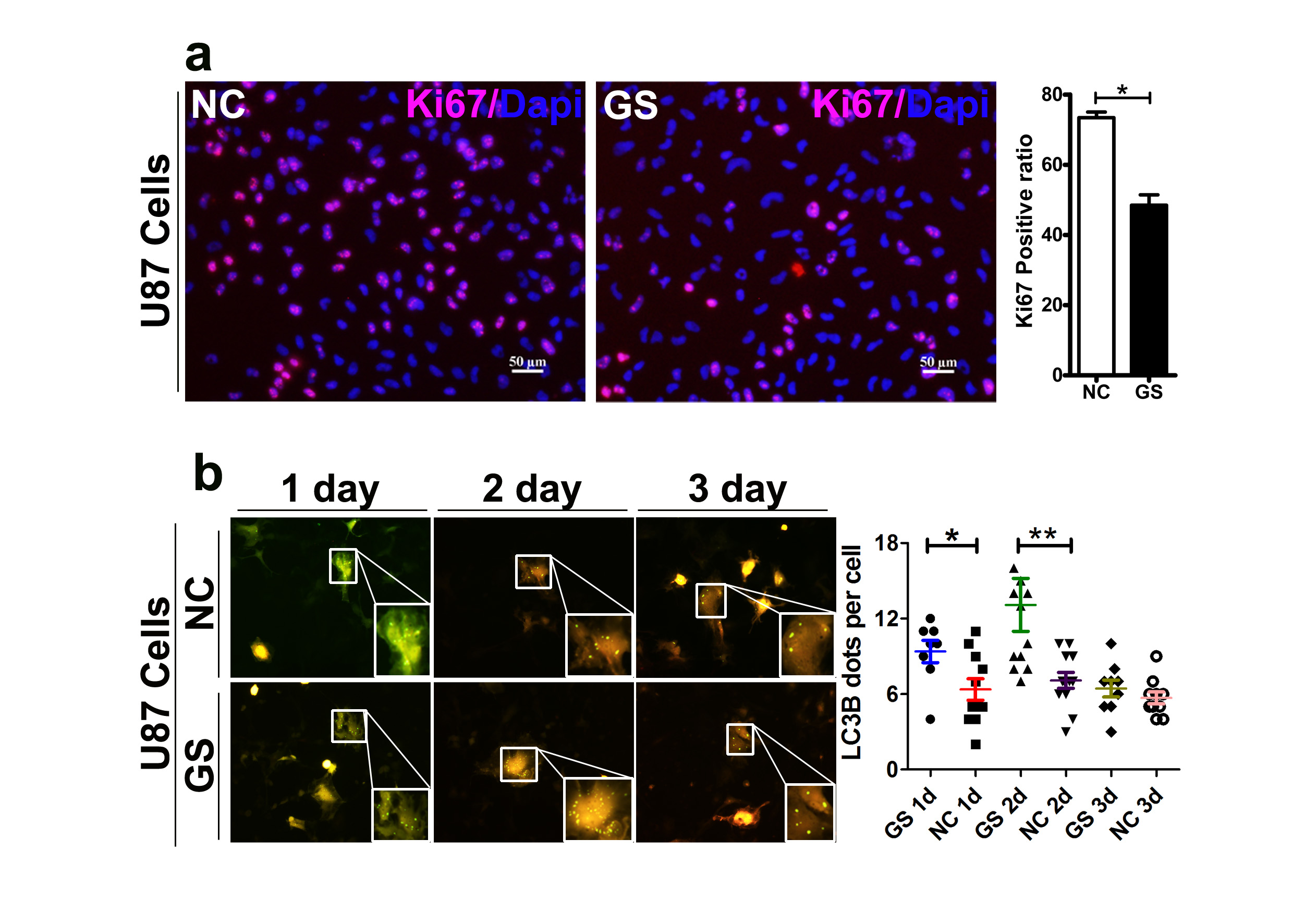
Supplementary Figure 1. Glucose starvation induces glioblastoma cells to exit cell cycle, enter quiescence and up-regulates autophagy.**

(**a**)There was a 20% decrease in Ki67^+^ proliferating cells with glucose starvation, compared to that with normal condition (**P*<0.05).

(**b**)Glucose starvation up-regulated autophagy as determined by the AAV-mRFP-GFP-LC3B reporter. The formation of autophagosomes representing autophagic activity was identified by yellow puncta containing both GFP and RFP signals. Quantification showed there were significantly more yellow puncta with glucose starvation (lower panels) than that with normal condition (upper panels, starting from the second day (**P*<0.05, ***P*<0.01).


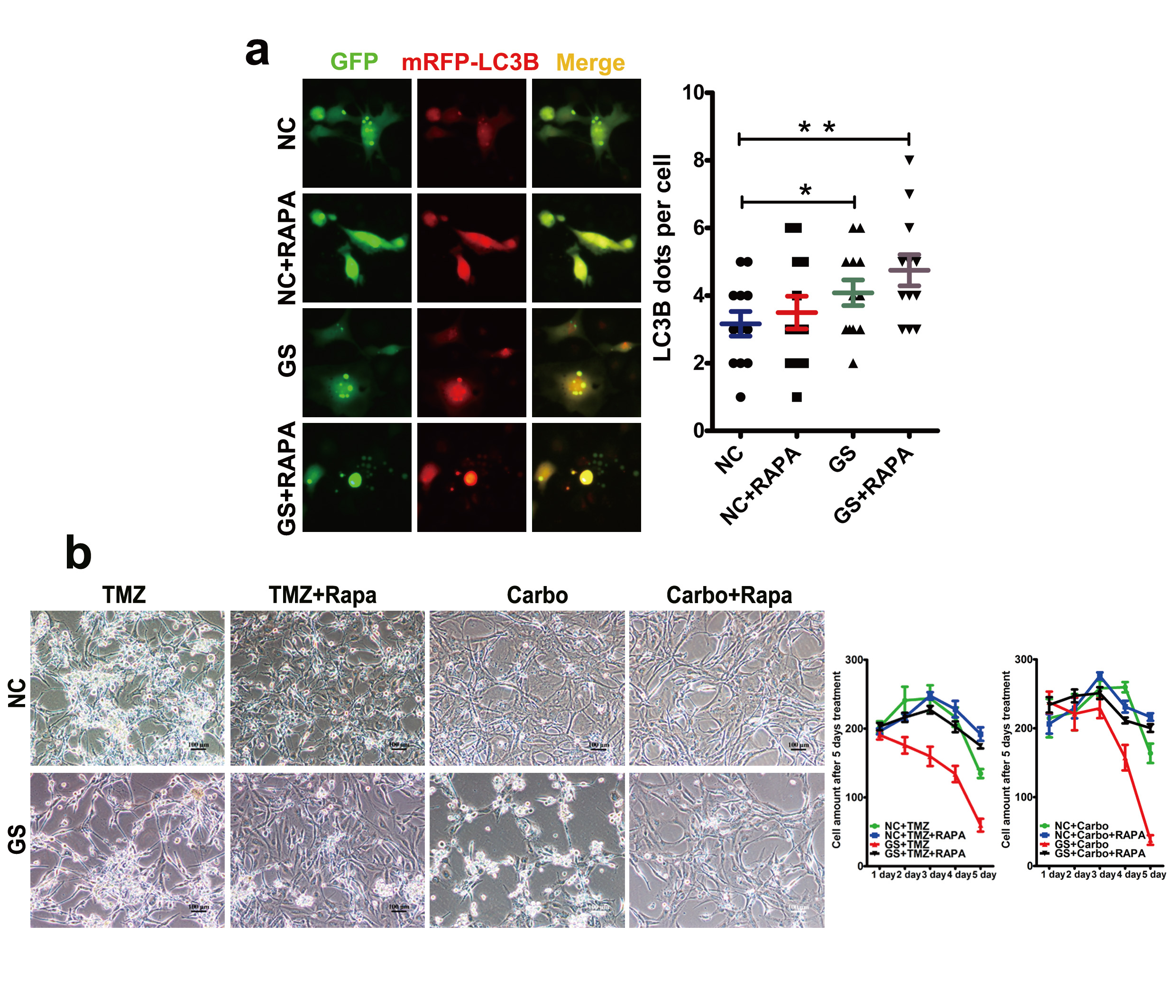


**Supplementary Figure 2. Autophagy promotes glioblastoma cell quiescence, incurring chemoresistance.**

(**a**)Rapamycin (RAPA) enhanced autophagy (lower panels) determined by AAV-mRFP-GFP-LC3B reporter (**P*<0.05, ***P*<0.01). Under normal (upper panels) and glucose starvation condition, there were more yellow puncta with rapamycin than that without the treatment. Combination of rapamycin and glucose starvation induced more formation of autophagosomes (***P*<0.01). In particular, there were significantly more cells of high autophagic activity in the combined treatment group than any of the other treatment ones.

(**b**)Rapamycin desensitized glucose-starved GBM cells to chemotherapeutic drugs and promoted their survival. Treatment with either temozolomide (TMZ) or carboplatin (Carbo) induced over 50% and80%GBM cell death with normal and glucose starvation conditions, respectively. Although it had little survival effect under normal condition, rapamycin dramatically reduced the cytotoxicity and rescued cell survival under glucose starvation condition. In the combined group, over 50% GBM cells survived, a 2-fold increase compared to that with the glucose starvation alone group.

**
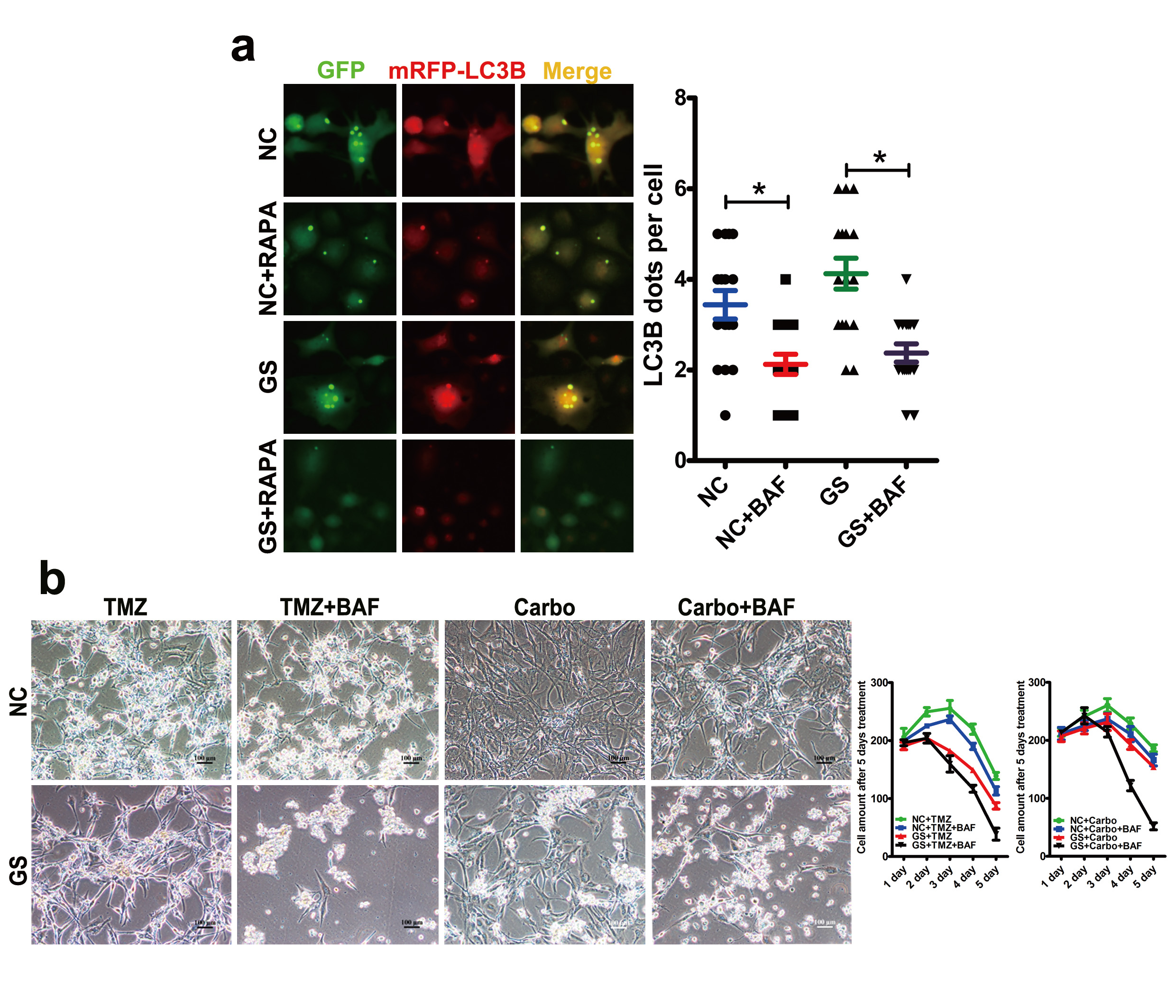
Supplementary Figure 3.** Autophagy inhibition alleviates chemoresistance of glioblastoma cells.

(**a**) Bafilomycin A1 (BAF) inhibited autophagy (lower panels) determined by the AAV-mRFP-GFP-LC3B reporter (**P*<0.05, ***P*<0.01). Under normal (upper panels) and glucose starvation condition, there were significantly less yellow puncta with bafilomycin A1 than that without the treatment (**P*<0.05, ***P*<0.01).

(**b**) Bafilomycin A1 (BAF) further sensitized glucose-starved GBM cells to chemotherapeutic drugs. The bafilomycin A1 enhanced the cytotoxicity with both normal and glucose starvation conditions. In particular, it effectively killed the subsets of cells that otherwise would have had entered quiescence, escaping from the chemotherapy drugs under the glucose starvation condition. As well, autophagy inhibition rendered the cells die faster and earlier.


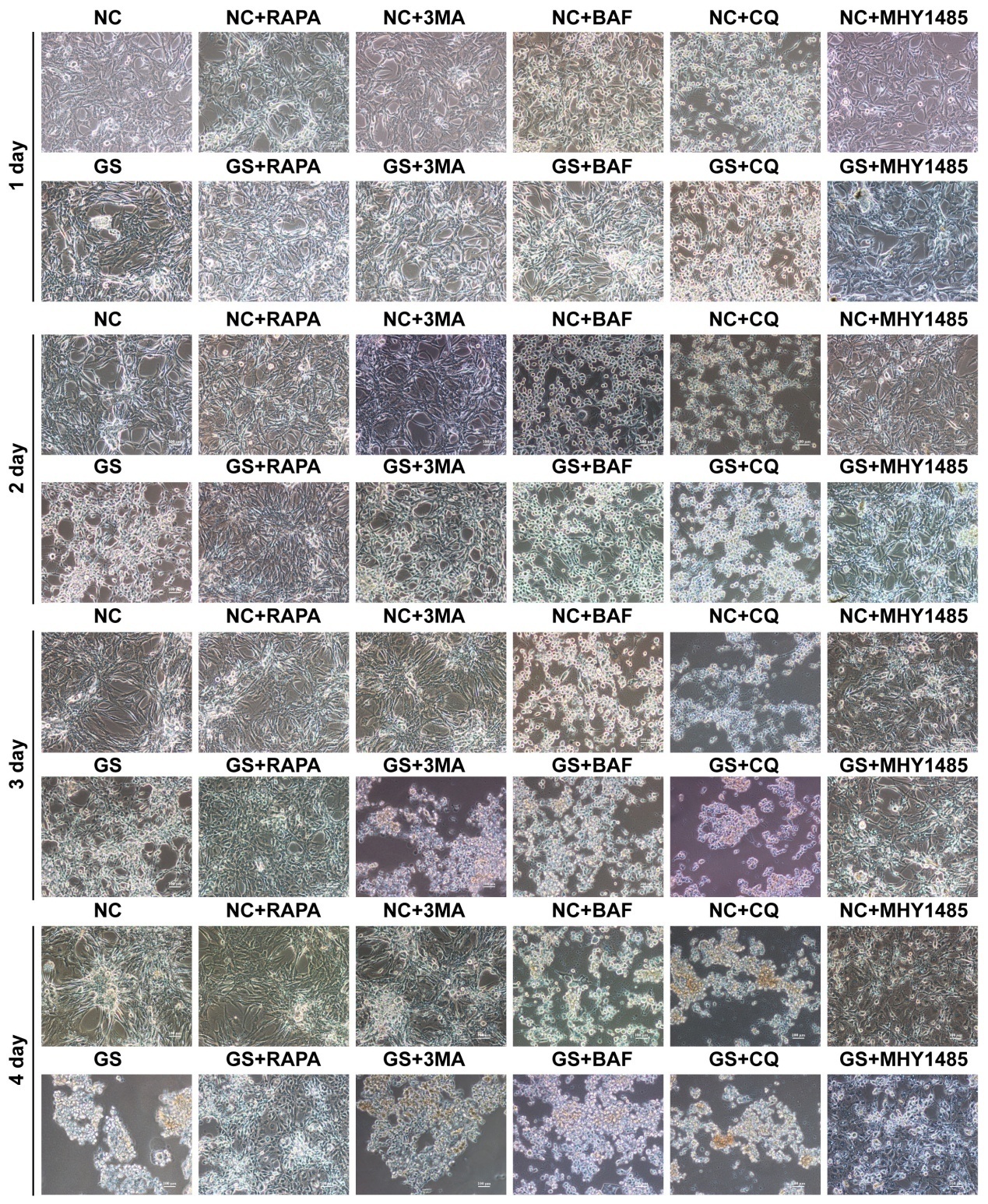


**Supplementary Figure 4.** Autophagy inhibitor 3-methyladenine (3-MA), bafilomycin A1 (BAF) and hydroxychloroquine (CQ) induced more cell death under both normal and glucose starvation condition especially could sensitize glucose starvation induced quiescent U87 cells to chemotherapeutic drugs. However, to our initial surprise, a fourth autophagy inhibitor MHY1485 failed to display similar function.

**
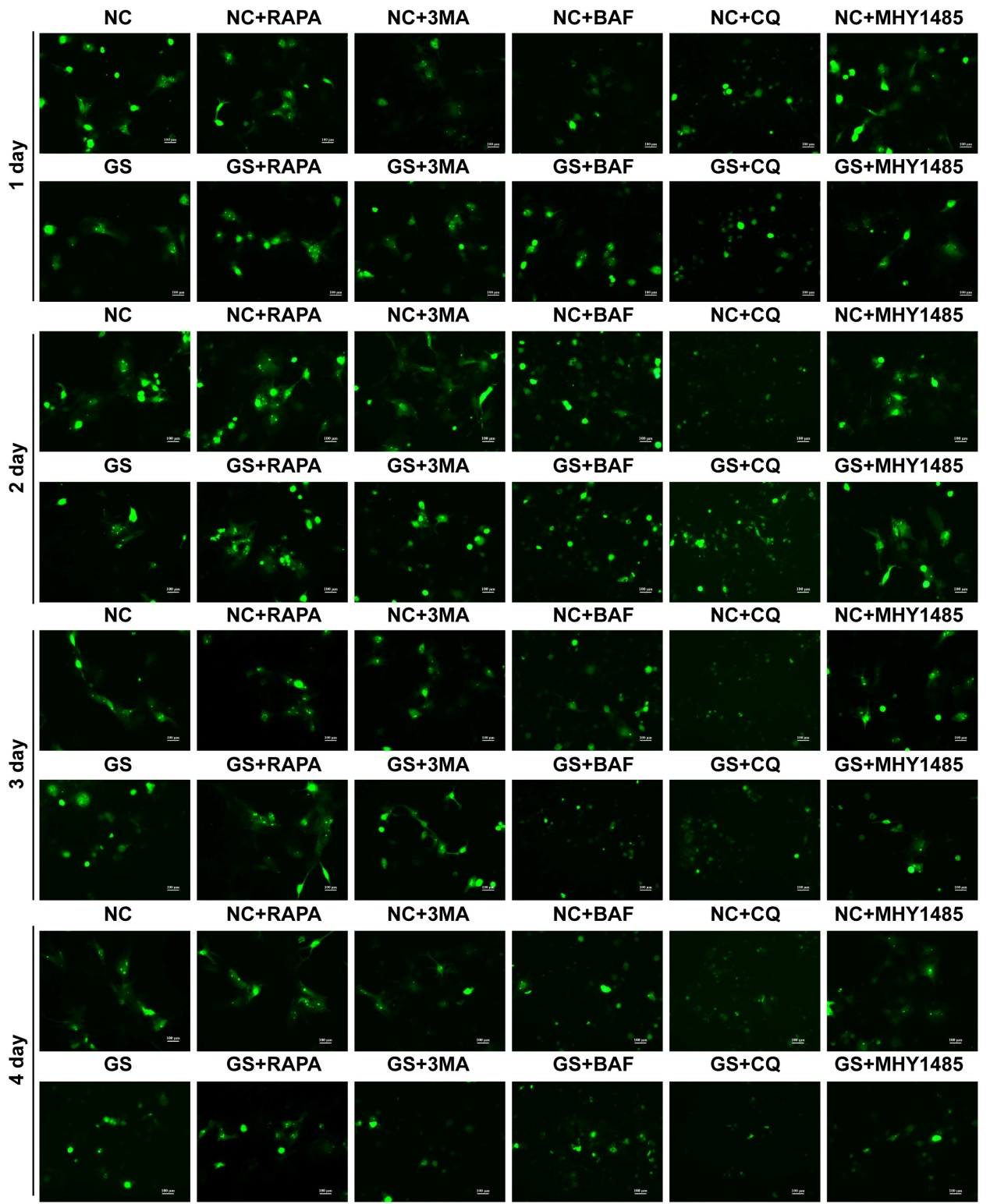
Supplementary Figure 5.** As determined by the mRFP-GFP-LC3B reporter, autophagic activity was significantly inhibited by autophagy inhibitor 3-methyladenine (3-MA), bafilomycin A1 (BAF) and hydroxychloroquine (CQ) under both normal and glucose starvation conditions. In contrast, MHY1485 could not repress the autophagic activity of GBM cells, explaining the puzzling observation thatMHY1485 failed to sensitize GBM cells to chemotherapeutic drugs.

**Supplementary Table 1 GO term analysis of DEGs down-regulated by autophagy**

| GO Component | Detail |
| --- | --- |
| Biological Process | Programmed necrotic cell death |
|  | Positive regulation of mitotic cell cycle |
|  | Cell division |
|  | Positive regulation of cell cycle |
|  | Microtubule cytoskeleton organization |
|  | G2/M transition of mitotic cell cycle |
|  | Apoptotic mitochondrial changes |
|  | Glucose homeostasis |
|  | Regulation of macroautophagy |
|  | Negative regulation of cell migration |
|  | Apoptotic signaling pathway |
|  | Cellular response to amino acid stimulus |
|  | Mitotic chromosome condensation |
|  | Intrinsic apoptotic pathway in response to oxidative stress |
|  | DNA strand elongation involved in DNA replication |
|  | Mitotic nuclear division |
|  | ATP metabolic process |
|  | Necroptotic process |
|  | Positive regulation of release of cytochrome c |
|  | Negative regulation of ROS metabolic process |
|  | Positive regulation of ATPase activity |
|  | Regulation of mitochondrial membrane permeability |
|  | Regulation of cell cycle |
|  | Positive regulation of apoptotic process |
|  | Glycolytic process |
|  | G1/S transition of mitotic cell cycle |
|  | Apoptotic process |
|  | DNA damage response |
|  | Anaphase-promoting complex-dependent catabolic process |
| MolecularFunction | Catalytic activity |
|  | L-aspartate transmembrane transporter activity |
|  | Microtubule binding |
|  | Protein phosphatase binding |
|  | GDP binding |
|  | Hydrogen ion transmembrane transporter activity |
|  | ATPase binding |
|  | Double-stranded DNA binding |
|  | Chaperone binding |
|  | GTP binding |
|  | Glucose binding |
|  | SH3/SH2 adaptor activity |
|  | Ubiquitin binding |
|  | Cysteine-type endopeptidase activity |
|  | Enzyme binding |
|  | GTPase activity |
|  | Hydrolase activity |
|  | Zinc ion binding |
|  | ATPase activator activity |
|  | Ubiquitin-protein transferase activity |
|  | Threonine-type endopeptidase activity |
|  | Ubiquitin protein ligase binding |
|  | ATP binding |
|  | Protein kinase binding |

**Supplementary Table 2 GO term analysis of DEGs up-regulated by autophagy**

| GO Components | Pathways |
| --- | --- |
| Biological Process | Cellular response to glucose starvation |
|  | Mitophagy |
|  | Glutamine metabolic process |
|  | Vasculogenesis |
|  | Negative regulation of cell proliferation |
|  | Autophagosome assembly |
|  | Apoptotic cell clearance |
|  | Macroautophagy |
|  | Cell migration |
|  | Cell-cell adhesion |
|  | Autophagy |
|  | Angiogenesis |
|  | Cell cycle arrest |
|  | Negative regulation of extrinsic apoptotic signaling pathway |
|  | Extracellular matrix organization |
|  | Positive regulation of cell migration |
|  | Protein autophosphorylation |
|  | Negative regulation of apoptotic process |
|  | Cytoplasmic translation |
| Molecular Function | Extracellular matrix binding |
|  | Insulin-like growth factor binding |
|  | Protein C-terminus binding |
|  | Rho GTPase binding |
|  | Transcription corepressor activity |
|  | Platelet-derived growth factor receptor binding |
|  | Rac GTPase binding |
|  | Protein kinase binding |
|  | Histone acetyltransferase binding |
|  | Receptor signaling protein serine/threonine kinase activity |
|  | ATP binding |
|  | Transforming growth factor beta binding |
|  | Chromatin binding |
|  | Transcription factor binding |
|  | Protein serine/threonine kinase activity |
|  | Sequence-specific DNA binding |
|  | RNA binding |
|  | Protein binding |

**Supplementary Table 3 KEGG pathwayanalysis**

| Type | Pathway |
| --- | --- |
| KEGG pathways analysis of DEGs down-regulated by autophagy | Central carbon metabolism in cancer |
|  | Other glycan degradation |
|  | Fc gamma R-mediated phagocytosis |
|  | Phosphatidylinositol signaling system |
|  | Endocytosis |
|  | Regulation of actin cytoskeleton |
|  | Ras signaling pathway |
|  | Toll-like receptor signaling pathway |
|  | FoxO signaling pathway |
|  | Oxidative phosphorylation |
|  | Glycolysis / Gluconeogenesis |
|  | Protein processing in endoplasmic reticulum |
|  | ECM-receptor interaction |
|  | Metabolic pathways |
|  | HIF-1 signaling pathway |
|  | Small cell lung cancer |
|  | Lysosome |
|  | DNA replication |
|  | PI3K-Akt signaling pathway |
|  | Mismatch repair |
|  | Phagosome |
|  | Carbon metabolism |
|  | Proteasome |
| KEGG pathways analysis of DEGs up-regulated by autophagy | Wnt signaling pathway |
|  | Non-small cell lung cancer |
|  | Acute myeloid leukemia |
|  | Bladder cancer |
|  | Rap1 signaling pathway |
|  | mTOR signaling pathway |
|  | Glioma |
|  | Melanoma |
|  | MicroRNAs in cancer |
|  | Alanine, aspartate and glutamate metabolism |
|  | Cell cycle |
|  | Colorectal cancer |
|  | MAPK signaling pathway |
|  | Prostate cancer |
|  | Pancreatic cancer |
|  | Pathways in cancer |
|  | AMPK signaling pathway |
|  | Chronic myeloid leukemia |
|  | PI3K-Akt signaling pathway |
|  | Thyroid hormone signaling pathway |
|  | Adherens junction |
|  | Hippo signaling pathway |
|  | FoxO signaling pathway |
|  | p53 signaling pathway |
|  | TGF-beta signaling pathway |
|  | Proteoglycans in cancer |

**Supplementary Table 4 DEG enriched in interested pathways**

| Pathways | Genes(Red: down-regulated genes; Black: up-regulated genes) |
| --- | --- |
| POSITIVE REGULATION OF APOPTOSIS | *CADM1, PAK6, PAK1, CUL1, PIM2, PRKCD, FAIM2, DPF1, EEF1A2, MLLT11, GJA1, NFKB1, PTEN, SHB, MYD88, NOD1, CASP7, CASP1, SGK1, BCL2L13, CTSC, TNFAIP3, TMEM214, PML, XAF1, PHLDA2, TRAF4, PHLDA1, STAT1, BRCA1, SEMA6A, PLSCR1, RPS6KA1, IRF1, DRAM1, ITGB3BP, RHOB, FAS, CASP2, MELK, STK17A, APAF1, TLR3, PTN, VAV3, NCSTN, ITGA6, ADM, RASSF2, ID3, NGFR* |
| NEGATIVE REGULATION OF APOPTOSIS | *ARNT2, ERBB4, FHL2, PLK2, IL6ST, STAT5B, ANKLE2, PDCD4, AKT1, RPS3A, TPT1, FAM129B, DDAH2, NQO1, MYC, CYR61, KIF14, TP53, PIM1, FLNA, MAP4K4, NME2, TXNDC5, HIPK3, THOC6, PDGFRB, NAIP, UBA52, GSTP1, UNG, PRKDC, PHIP, XBP1, TGM2, THBS1, CSF1R, ALMS1, RPS6, SIRT1, BFAR, HDAC2, UCP2, CD59, ZMYND11, AR, ITGAV, TGFBR1, GCLM, ACVR1* |
| UPREGULATION OF AUTOPHAGY | *CHMP3, GABARAPL1, ULK1, ARSB, HMGB1, STAM2, XBP1, SNF8, TBC1D5, CTSD, WIPI1, ATG2B, ATG14, TOMM7, NBR1, UBA52, RPS27A* |
| PATHWAY IN CANCER | *ARNT2, GNAI1, TRAF3, MET, ADCY7, TGFB2, AKT1, RHOA, MYC, CTBP2, RXRB, TP53, CCND1, PDGFRB, GNG12, ITGAV, RUNX1, CSF1R, EPAS1, FZD2, CBLB, HDAC2, BAX* |
| CELL CYCLE ARREST | *PPM1A, ING4, HBP1, SKIL, THBS1, GAS6, TSC2, TGFB2, CDKN2D, MYC, TP53, ATM, RBL2, PRKDC, CDC25C, WEE1, CCND1, HDAC2, STAG1, NFE2L2* |
| CELL CYCLE | *MADD, PIM2, CCNA1, ENSA, OPTN，PHACTR4, KIAA0101, ARNTL, CLIC1, MYBL2, PTEN, STAT3, BAK1, IRF1, ID3, MX2, CCNB1, SKP2, CDC25A, CDC7, CDC6, DBF4B, MCM10, MCM4, MCM3, RPA1, TYMS, RRM2, PCNA, CDK2, RAB8A, AURKA, PPP1CB, MELK, PHLDA1* |
